# Two-channel models of medial and superior olive based on psychoacoustics

**DOI:** 10.1186/1471-2202-16-S1-P276

**Published:** 2015-12-18

**Authors:** Jaroslav Bouse, Vaclav Vencovsky

**Affiliations:** 1 Department of Radioelectronics, Faculty of Electrical Engineering, Czech Technical University in Prague, Prague, 162 27, Czech Republic; 2 Musical Acoustics Research Center, Academy of Performing Arts in Prague, Prague, 118 00, Czech Republic

## 

Two nuclei in each hemisphere, the medial superior olive (MSO) and the lateral superior olive (LSO), are responsible for decoding interaural time difference (ITD) and interaural level difference (ILD), respectively [1]. This allows us to localize sound sources in the horizontal plane.

Neurons in the MSO are usually modeled as coincidence detectors [1]. Two main approaches to model the MSO appeared during last decades. The most utilized approach (introduced by Jeffress [2]) uses a delay line together with coincidence detectors. The model assumes that the individual neurons in the MSO are sensitive to the different ITD. Another approach (introduced by von Békésy [3]) accounts for two channels in the brain competing with each other. The concept of two channel model was further developed by Dietz [4] (called rate-code model), and Pulkki and Hirvonen [5] (called count-comparison model), who incorporated new physiological findings. The LSO neurons are consistently reported as an Excitation-Inhibition type [1] and mainly ILD sensitive, thus it can be modeled by a simple subtracter.

We present phenomenological models mimicking the functions of the MSO and LSO. The models incorporate the latest physiological findings. However, the models were not designed to simulate responses of the neurons in the MSO and LSO. Instead, the models give data directly comparable with results of subjective lateralization experiments. To simplify the readability, we call these models according to the nuclei which they are mimicking, the MSO and the LSO model. As a front-end, a model of peripheral ear (taken from the literature) is utilized. The MSO model is Excitation-Inhibition type, in principle similar to the two channels, rate-code or the count-comparison models. The LSO model is Excitation-Inhibition type with a simple subtracting unit simulating the process inside the LSO. There is one MSO and LSO model for each hemisphere. Outputs from both hemispheres are compared following the two channels principle. Interaction between the MSO and the LSO model output is not yet possible in this implementation.

We compare the outputs of the designed models with the results of psychoacoustic experiments showing the lateralization of pure tones and narrow band noises. The data illustrating the lateralization of pure tones with ITD or ILD was reproduced from the literature [6]. A psychoacoustic experiment was conducted to measure the subjective lateralization of a narrowband noise (central frequency 380 and 760 Hz, bandwidth 1 ERB) with interaural phase or level differences. Seven and five normal hearing listeners participated in the case of interaural phase and level differences test, respectively. A good agreement between the model data and subjective lateralization was observed.
